# Streamlining standard bacteriophage methods for higher throughput

**DOI:** 10.1016/j.mex.2018.01.007

**Published:** 2018-01-31

**Authors:** Kathryn M. Kauffman, Martin F. Polz

**Affiliations:** a Department of Civil and Environmental Engineering, Massachusetts Institute of Technology, Cambridge, MA, 02141, USA; b The Center for Microbiome Informatics and Therapeutics, Massachusetts Institute of Technology, Cambridge, MA, 02141, USA

**Keywords:** Agar overlay, Agar overlay, Agar layer, Top agar, Bottom agar, Plaque, Virus, Phage, Isolation, Cultivation, Purification

## Abstract

A universal tool in the culture-based study of bacterial viruses (bacteriophages, or phages) is the agar overlay, which is used in the isolation of new viruses, and in their quantification and purification. Here, simple optimizations that increase efficiency and throughput in agar overlay based isolation and cultivation of virus-host systems are presented. The agar overlay is streamlined to minimize steps and materials. Serial purification of viruses from viral colonies (plaques) is optimized to eliminate steps by combining purification by serial re-streaking with the optimized agar overlay approach. Finally, recommendations are made for efficient archival and storage of virus plaques. In sum, this work presents:

• Tube-free Agar Overlays: rapid plaque assays with fewer steps and materials
• Molten Streaking for Singles: rapid tube-free serial purification of viruses
• Archiving Plaques: saving virus purification for later

## Graphical abstract

**Figure:**
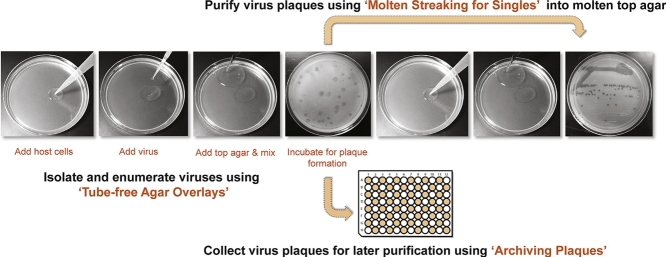

## Methods

The presented methods are host-system independent and thus do not specify media composition beyond the percent agar in top agar and bottom agar as these will be defined by the user. See the “Additional Information” section below for specific additional considerations that may affect recovery or detection of different groups of viruses.

## 1 Tube-free Agar Overlays: rapid plaque assays with fewer steps and materials

### 1.1 Background and applications

This protocol simplifies current approaches for agar overlay plaque assays by eliminating the use of tubes for premixing of agar, hosts, and viruses, in favor of pipetting each of these directly onto the bottom agar. The benefits of this approach include simplified experimental set-up, and reductions in preparation and clean-up times and material requirements. In addition, by eliminating tube-based steps, the duration of exposure of cells and viruses to heat is reduced and the need for potentially damaging vortexing or agitation [1] of virus-host mixtures is also eliminated. We note that Hershey et al. mentioned the possibility of such an approach in their 1943 description of the tube-based overlay procedure [2], stating: “A further slight improvement may be made by mixing the sample directly on the phage with only 3 mL 0.7% agar, but the mixing is difficult.” By using a lower percentage agar, which is advantageous for other reasons (see Additional information section, below), we find that this approach works very well with as low as 2 mL of top agar.

Using this streamlined, tube-free, plating method, >45 samples can readily be plated per hour from a common or pre-prepared virus stock, without the need for individual tubes of molten agar or multiple transfers of bacteria and virus. When deployed for isolation of novel viruses, or quantification of environmental viruses, this translates to a potential for screening hundreds of potential host strains per day using a single bottle of molten agar rather than hundreds of tubes. When used for routine bench assays, a single bottle of top agar can be used over multiple days by simply re-microwaving.

### 1.2 Preparation of materials

• **Host culture**: This procedure works well with 100 uL of overnight host culture (∼10^9^ cfu mL^−1^) for each standard size (100 mm) petri dish.
• **Virus material**:
  ○ For ***Isolation of Viruses*** the appropriate stock material can include any potential virus source, for example: an iron-chloride flocculate of filtered seawater, resuspended in oxalate solution [[3], [4]]; a PEG-precipitate of sewage supernatant; or soil or stool resuspended in buffer and then pelleted or filtered to remove cells. The total volume needed per plate depends on the concentration of viruses in the stock material but the agar overlay procedure can accommodate up to several hundred microliters of material; at higher volumes of stock material (greater ∼ 100uL) it is recommended that the volume of host and top agar be increased proportionately, up to 3 mL top agar.
  ○ For ***Quantification of Virus Titer by Direct Plating*** the appropriate stock material is a 10-fold dilution series of the virus stock in buffer or media, with a recommended plating volume of ≥10 uL per plate.
• **Bottom agar plates**: Prepare media containing 1.0% agar ('bottom agar') in a glass bottle or flask with a stirbar and sterilize, pour 25 mL per standard size petri dish, and allow to solidify.
• **Top agar bottle**: Prepare media containing 0.3% agar ('0.3% top agar') in a glass bottle with a PTFE-coated stirbar and sterilize; though each plate will require only 2 mL of top agar, volumes of up to 500 mL can be prepared and re-used across multiple days of plating.

### 1.3 Preparation of the top agar in beaker-waterbath

**Fig. 1:**
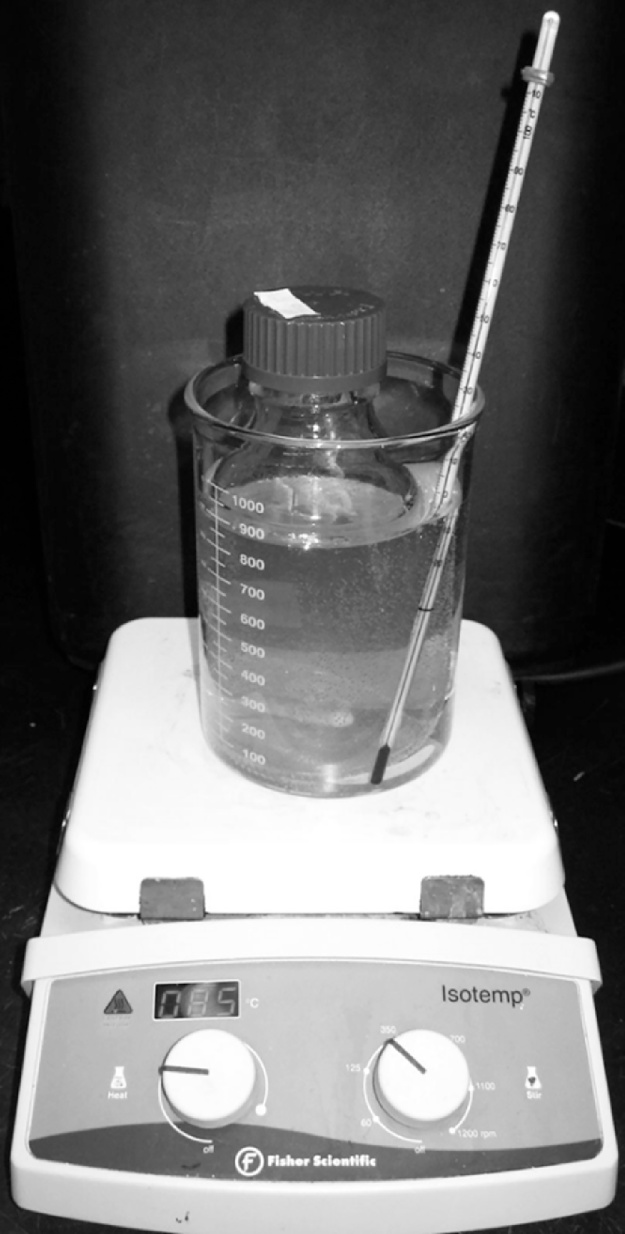
Top agar bottle equilibrated in beaker-waterbath.
• **Melt the top agar**: Place the beaker-waterbath with the top agar bottle into a microwave and cook without boil-over until thoroughly melted.
  ○ *Notes*:
    ○ Ensure that the lid of the top agar bottle is slightly loose to allow for venting during microwaving.
    ○ It is exceedingly important to achieve a 'smooth melt' of the top agar to ensure that plaques will form and be visible. Media composition affects the time it takes to achieve a 'smooth melt', however a general approach is as follows: start by melting the top agar in a microwave for several 5-min cycles at low% power, once the top agar appears nearly completely melted increase the% power and cook until the top agar comes to a boil, observe the top agar during high power cooking to prevent boil-over, bring to a boil 3 times.
• **Equilibrate the top agar to 50**–**52** **°C**: Place the beaker-waterbath containing the bottle of molten top agar and stirbar onto a hot plate and activate gentle stirring, place a thermometer into the beaker-waterbath and leave it there to wait for equilibration to 50–52 °C.
  • *Note*: This will require setting the heat block to a temperature greater than the target temperature, for example up to 85 °C, but this is dependent on specific heat block models and must be determined by the user.

• **Prepare a beaker-waterbath**: Place bottle of top agar into a glass beaker and add water to the beaker up to the level of the top agar in the bottle; for example, a 500 mL glass bottle into a 1 L beaker (final set up shown in Fig. 1)

### 1.4 Procedure for Tube-free Agar Overlays

**Fig. 2:**
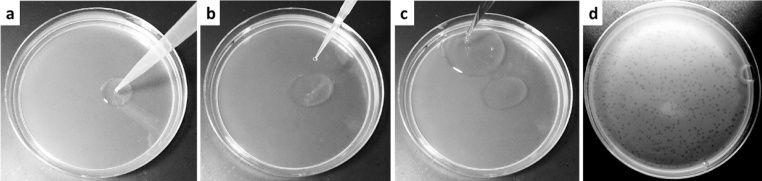
Steps in the Tube-free Agar Overlay. First, the host culture is pipetted directly onto the bottom agar (a); second, the viruses are pipetted directly into the drop of host culture (b); third, molten top agar is pipetted from the top agar bottle directly onto the bottom agar adjacent to the drop of host and virus (c); the plate is then picked up and swirled vigorously but briefly to mix the bacteria, virus, and agar, and to form an even overlay. Following incubation, lawns of susceptible hosts will show formation of evenly distributed plaques (d).
  ○ Pipette 10–100 uL of virus-containing solution directly into the host droplet on the bottom agar (Fig. 2b).
  ○ Remove the lid of the top agar bottle briefly to pipette out 2 mL of molten top agar using either a 5 mL pipette or a serological pipette, and sterile technique.
  ○ Pipette the 2 mL of molten top agar from the bottle directly onto the bottom agar next to the droplet of hosts and viruses (Fig. 2c).
  ○ Swirl the plate vigorously but briefly to mix the bacteria and virus and molten top agar and to spread it across the plate.
  ○ Leave the plate on the bench, top agar side up, for at least 20 min to allow the agar to completely solidify, then place in desired incubation conditions and monitor for plaque formation (Fig. 2d).
  ○ *Notes*:
    ○ Failure to achieve satisfactory lawns is often due to either: 1) insufficiently melted top agar, which gives rise to matte lawns instead of glossy smooth lawns, and 2) taking too long to swirl the agar thus allowing it to cool down and set before the mixing is completed.
    ○ As in all plating assays, it is recommended that control plates be included to ensure that: 1) the agar overlay is contaminant free at the start and finish of the experiment (include only top agar in these controls), 2) the bacterial stock is virus-free (include only bacteria and top agar in thee controls), and 3) the virus stock is not contaminated with cells (include only virus stock and top agar in these controls).

• **Agar overlay**:
  ○ Pipette 100 uL of overnight host culture directly onto the bottom agar (Fig. 2a).

### 1.5 Example of use

The Tube-free Agar Overlay is useful as a simple and efficient standard approach for all basic bench protocols requiring agar overlays. We note that a comparison of tube-free and tube-based methods for determination of titers, for example, shows no significant differences in estimates of titer between the two methods (Table 1). This approach also affords considerable advantage in any studies or assays requiring plating of large numbers of overlays, such as studies of the ecology of environmental virus-host interactions, where even the most closely related bacteria recovered from a sample may have distinct genotypes [[5], [6]] and thus virus susceptibilities. For example, the Tube-free Agar Overlay was used in a study of the ecology of virus-host interactions in the marine environment [7] to perform plaque assays on >1300 marine bacterial isolates using iron oxalate seawater concentrates [7].

**Table 1:** Comparison of PFU mL^−1^ estimates for three virus stocks with tube and tube-free plating approaches. Values shown are mean and percent standard deviation, n = 3.

Virus	Tube-free (PFU mL^−1^)		Tube(PFU mL^−1^)		Paired *t*-test *(P sig at 0.017 with Bonferroni Correction)*	
*Vibrio* phage 12G01	1.60 × 10^10^	±1%	1.52 × 10^10^	±11%	t_0.05(2),(2)_ = 0.88	P = 0.471
*Vibrio* phage Jenny	1.41 × 10^11^	±3%	1.57 × 10^11^	±3%	t_0.05(2),(2)_ = −6.80	P = 0.021
*Vibrio* phage Al	1.39 × 10^11^	±9%	1.52 × 10^11^	±11%	t_0.05(2),(2)_ = −1.53	P = 0.266

## 2 Molten Streaking for Singles: rapid serial purification of viruses from plaques

### 2.1 Background and applications

This protocol presents a simplified approach for re-streaking based purification of viral strains directly from plaques using the Tube-free Agar Overlay method. Viruses are ‘picked' directly from source plaques with a toothpick and streaked into still-molten agar overlays prepared using the Tube-free Agar Overlay approach in a manner directly analogous to re-streaking based colony purification of bacteria. This approach is similar in principle to direct streaking for singles onto bottom agar [[1], [8], [9]] but eliminates the need for tubes, eliminates the need to spread molten top agar over the virus streak – which may lead to resuspension and mixing off the streak, and reduces the amount of top agar needed as the overlay is vigorously spread before the viruses are added. This Molten Streaking for Singles approach requires less time and material than approaches based on plating of dilutions or streaking onto bottom agar and yields equivalent purification of viruses.

### 2.2 Preparation of materials

• **Host culture**: This procedure works well with 100 uL of overnight host culture (∼10^9^ cfu mL^−1^) for each standard size (100 mm) petri dish.
• **Virus material**:
  ○ For ***Serial Purification of Viruses*** the starting material for the first streak is any material enriched for viruses of interest, such as a previous agar overlay containing a plaque to be purified, or an archived plaque eluate (as described in Section 3.3). The starting material for the second and third purification streaks is a well-separated plaque from the previous streak.
• **Bottom agar plates**: Prepare media containing 1.0% agar ('bottom agar') in a glass bottle or flask with a stirbar and sterilize, pour 25 mL per standard size petri dish, and allow to solidify.
• **Top agar bottle**: Prepare media containing 0.3% agar ('0.3% top agar') in a glass bottle with a PTFE-coated stirbar and sterilize; though each plate will require only 2 mL of top agar, volumes of up to 500 mL can be prepared and re-used across multiple days of plating.

### 2.3 Preparation of the top agar in beaker-waterbath

• Prepare top agar in beaker-waterbath, as described in Section 1.3.

### 2.4 Procedure for Molten Streaking for Singles

**Fig. 3:**
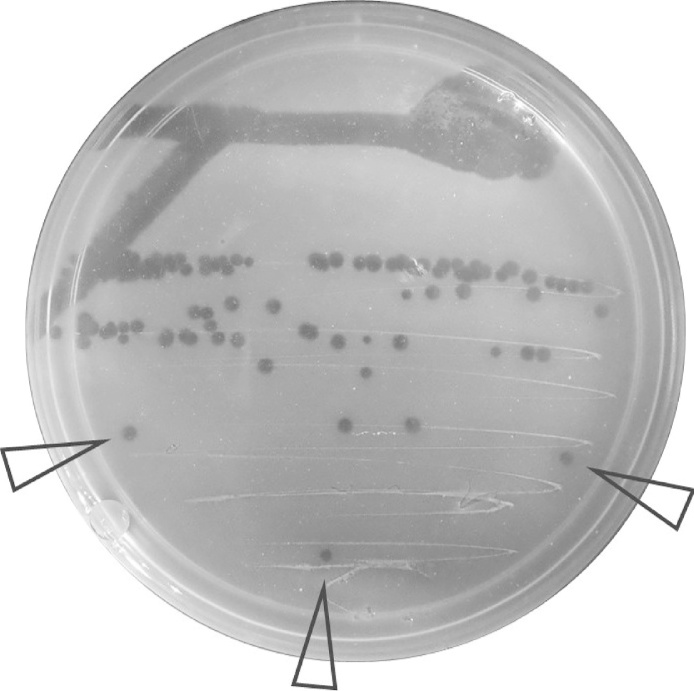
Formation of plaques in agar overlay host lawn following Molten Streaking for Singles. Arrows indicate well-separated plaques appropriate for selection for further purification.
• **Streaks 2 & 3 (Days 2 & 3)**: Once plaques appear on the first plate, use this plate as a source, insert a sterile toothpick into a single plaque near the terminus of the streak and repeat the streaking for singles approach for the desired number of purifications. Single plaques arising from the final streak can be picked and archived, or used to generate large-scale liquid or plate lysates of the purified virus.
  • Note: It is common practice to consider plaques generated from the third streak as “purified”. When deciding on number of serial purifications consider that use of fewer serial passages may result in recovery of mixed stocks of viruses but may also minimize changes in viruses resulting from adaptation or selection on the host during passage.

• **Prepare the molten agar overlay (Day 1)**: Have all materials ready to advance to Streak 1 step before starting this step as the lawn cannot be allowed to solidify before the streak
  ○ Pipette 100uL of overnight host culture directly onto the bottom agar.
  ○ Pipette 2 mL of molten top agar from the bottle directly to the bottom agar next to the hosts using either a 5 mL pipette or a serological pipette.
  ○ Swirl vigorously but briefly to mix the bacteria and molten top agar and spread it across the plate.
• **Streak 1:** Streak viruses from the source plate directly into the still-molten top agar (Video 1) such that when the host lawn forms it will contain single colonies; it is necessary to do this swiftly such that streaking is completed before the agar solidifies.
  • Insert a sterile toothpick (or pipette tip) into the source plaque or solution.
    ○ Note: When isolating from a plaque, ensure that the plaque is well separated from other plaques to prevent contamination from viruses diffusing from neighboring infections, which may occur even once plaques have stopped growing.
  • Swirl the toothpick into a small area of still-molten agar overlay.
  • Use a second toothpick to make one stroke through the area where the first toothpick was touched.
  • Use a third toothpick to make a repeating Z-stroke through the still-molten top agar, first passing once through the streak from the second toothpick.
• **Allow agar overlay to solidify**: Leave the plate on the bench, top agar side up, for at least 20 min to allow the agar to completely solidify, then place under desired incubation conditions and monitor for plaque formation (Fig. 3).

### 2.5 Examples of use

The Molten Streaking for Singles approach is useful as a simple and efficient standard approach for basic bench protocols requiring serial purification of viruses or recovery from stocks of unknown titer (as, for example, from archived plaques and plaque-eluates as described in Section 3). This approach affords considerable advantage in any studies or assays requiring purification of large numbers of viruses. For example, in a study of the diversity and host range properties of marine bacterial viruses, >250 viruses were isolated from screening overlays (Section 1.5), archived using the Archiving Plaques approach (Section 3), and then later purified and amplified for genome sequencing using triple purification with the Molten Streaking for Singles approach [7].

## 3 Archiving Plaques: saving virus purification for later

### 3.1 Background and applications

In plaque-assay based viral discovery many more plaques are often generated than can be examined at any one time, it is therefore of value to be able to archive plaques for future investigation. To address the potential for differences among viruses in tolerance to storage, this protocol provides large-scale archival approaches that include storage of picked plaques at both 4 °C and −20 °C or −80 °C. We note however, that viruses differ in their sensitivity to storage conditions [[10], [11]] and, though we describe methods that include multiple conditions, we highlight here that including additional approaches, such as storage in infected cells [12], is likely to increase total proportion of recovered viruses in large collections.

### 3.2 Preparation of materials

The described procedures are of greatest utility when the number of plaques to be archived is on the order of several hundreds.

• **Virus material**:Agar overlay plates with plaques to be archived
• **Cataloging spreadsheet**, for an example see Supplementary file 1
• **Archival and filtration materials**
  ○ Sterile host growth media (250 uL per plaque)
  ○ Sterile 50% glycerol solution (50:50 glycerol:host growth media; 125 uL per plaque)
  ○ Sterile adhesive aluminum foil 96-well plate covers (VWR 60941-076)
  ○ When opting for centrifugation-based filtration:
    ○ Users will need to ensure compatibility of selected filter-bottom plates and receiver plates. The below items are an example of compatible components.
    ○ 96-well polypropylene microplates for collection and as filtration receivers (Greiner Bio-One #651261); requires two 96-well plates per every 48 plaques.
    ○ 96-well filter-bottom plates, 0.22 um (EMD Millipore MSGVS2210) or 0.45 um (EMD Millipore MSHVS4510); requires one 96-well plate per every 48 plaques.
    ○ Centrifuge that can accommodate 96-well plates
  ○ When opting instead for vacuum-based filtration:
    • Users will need to ensure compatibility of selected manifold, filter-bottom plates, and receiver plates. The below items are an example of compatible components.
    • 96-well polypropylene microplates for collection and as filtration receivers (Greiner Bio-One #651261); requires two 96-well plates per every 48 plaques.
    • 96-well filter-bottom plates, 0.22 um (EMD Millipore MSGVS2210) or 0.45 um (EMD Millipore MSHVS4510); requires one 96-well plate per every 48 plaques.
    • MultiScreenHTS Vacuum Manifold for 96-well plate format (EMD Millipore MSVMHTS00)
    • Vacuum pump

### 3.3 Procedure for Archiving Plaques

In cases where there are hundreds of plaques of interest it is optimal to divide the tasks of cataloging, collecting, and final processing over three consecutive days.

• **Cataloging Plaques (Day 1)**:
  ○ Mark all plaques to be archived by writing a number adjacent to the plaque on the bottom of the petri dish.
  ○ Log all plaque properties of interest into an archival spreadsheet, such as the example provided in Supplementary file 1. Properties of interest might include: day plaque was first detected, size, and appearance.
• **Collecting Plaque Plugs (Day 2)**:
  • Prepare 96-well plates for receiving plaque plugs by aliquoting 250 uL of host growth media (or desired buffer) into 48 alternating wells. Note that alternating wells are used to minimize potential for cross-contamination between stocks.
  • For each plaque, collect a plaque plug as follows:
    ○ Hold a 1 mL pipette-tip by hand and use it to bore through the plaque to the bottom of the petri dish.
    ○ Twist the tip at the bottom of the dish to completely sever the connection between the plug and the rest of the agar.
    ○ Pull the pipette tip free from the agar and insert the delivery end into the designated well in the 96-well plate, as defined in the cataloging spreadsheet.
    ○ Press down on the opening at the top of the pipette tip with a finger to create sufficient air pressure to expel the agar plug containing the plaque into the media in the well. This may require several presses. Confirm that the plug is dislodged and in the media.
  • Once all plaque plugs have been harvested seal the 96-well plate with sterile adhesive aluminum covers and store at 4 °C overnight to allow the virions to elute from the plugs.
• **Processing Plaque Plugs (Day 3)**
  • Prepare a filter-bottom plate by removing it from its package and placing it on top of a receiver plate snugly. For centrifugation-based approach use lab tape to seal the filter plate snugly to the receiver plate.
  • Using a multichannel pipette, transfer 125 uL from each well of the storage plate to the filter-bottom plate. Though it is preferable to avoid collecting the plaque-plug in the pipette tip during transfer it ultimately does not matter as the virions are expected to have been eluted into the media overnight.
  • Filter the plug eluate through the filter plate, either:
    ○ By vacuum filtration using a vacuum manifold, or
    ○ By centrifugal filtration using a centrifuge that can accommodate filter plates, spin up to 30 min at max speed ≤5000 rcf to collect maximum filtrate.
  • Remove and discard the filter-bottom plate from the receiver plate and cover the receiver plate with a sterile adhesive aluminum foil cover. This virus stock material is cell-free and can be stored at 4 °C.
  • Add 125 uL of 50% glycerol solution to the 125 uL remaining in the original plaque-eluate plate. This virus stock material contains cells and should be stored at −20 °C or −80 °C to prevent cell growth.
• **Recovering viruses from archives (future)**
  • Simply use the Molten Streaking for Singles approaches described above to prepare fresh plates of plaques from the archived plaque material.
    ○ *Notes*:
      ○ The concentration of viruses in the archived samples will vary depending on the initial number of viruses in the plaque and specific decay rate of each type of virus [13]. It is therefore recommended that screening of stored material to recover plaques begin with Molten Streaking for Singles from a 20 uL volume of the 4 °C archived sample. If the 4 °C stock does not yield plaques, repeat with a small mass of the frozen stock.
      ○ It is exceedingly important when working with 96-well plates of frozen stocks to ensure that they remain frozen to prevent loss of virus stocks that are sensitive to multiple freeze-thaw cycles.

### 3.4 Example of use

The Archiving Plaques approach affords considerable advantage when a study of viral diversity generates a larger number of plaques from precious limited sample material than can be purified or screened for desired properties immediately. Such cases include collaborative or time-series studies, where only a limited amount of material relevant to each study unit or time point is available and must suffice for all the interests of the investigation.

For example, in a study of the diversity and dynamics of marine bacterial viruses [7], >450 hosts were screened for sensitivity to co-occurring viruses (Section 1.5) at each of three time points in the context of larger well-characterized time series where there was limited virus concentrate from each day. The plating of material from these three time points generated thousands of plaques, representing an opportunity to gain insight into the ecological dynamics of virus activity by study of ecologically representative diversity of viral isolates, however it was not possible to process all of these together. Using the Archiving Plaques approach, it was possible to archive the plaques and return to the stocks later in time to initiate purification and amplification for genome sequencing and study, which also lead to the discovery of a proposed new family of bacterial viruses [14].

## Additional information

### Relevance

The study of virus isolates provides insight into the ecology and evolution of their hosts, and thus also for improved rational design of virus-based therapeutics (such as “phage therapy” [15]) and bioengineering tools [[16], [17], [18]]. The current dominance of unassignable “dark matter” [19] in viral metagenome sequence databases indicates however, that current culture collections do not adequately represent natural diversity, suggesting that much of the dynamics between viruses and their hosts remain poorly understood. Recovery of diverse culture collections of microbial viruses therefore still stands as a major avenue forward for improving fundamental mechanistic understanding of virus-host interactions, for classification of “dark matter”, and for the development of tools to precisely and efficiently manipulate microbial systems, which may require “cocktails” [20] of multiple viruses. With its simplicity and flexibility, the agar overlay offers a powerful approach for culture-based discovery and characterization of viruses and promises to continue on as a workhorse in the study of virus-host interactions for some time.

### Background

Felix d’Herelle’s observation of small clearings in bacterial lawns, first reported by him in 1917 [21], and his recognition that they represented killing by “invisible microbial antagonists” of bacteria, mark the beginning of the use of plaque assays in the study of viruses. The development of a method for improving plaque assays by incorporating viruses and hosts into a low density ‘top’ agar layered upon a higher density ‘bottom’ agar layer was described by André Gratia in 1936 [[22], [23]], and by Alfred Hershey et al. in 1943 [2]. In such methods the thin low density ‘top agar’ layer allows for growth of cells and diffusion of virus particles, and the ‘bottom agar’ provides a source of nutrients to cells in the top layer that sustains growth of hosts long enough for viruses to complete multiple cycles of replication and thereby form macroscopic plaques [24]. The infection of a bacterial cell by a single lytic virus can thus be observed as a zone of clearing, a plaque, in the confluent lawn of host bacteria. A description of the method was included in a report on bacteriophage methods by Mark Adams [[25], [26]] in 1950, where he referred to it as the “agar layer” method and described it as “in general use by practically all workers”. The 1959 vol Bacteriophages, written in large part by Mark Adams – but finished by others due to his death at the age of 44 in 1956 due to an infection – includes the agar overlay and is the most widely cited description of the method. Readers interested in early bacteriophage literature are also referred to Hansjürgen Raettig’s comprehensive summary [27] and index [28] of publications in the field from 1917 to 1956.

**Fig. 4:**
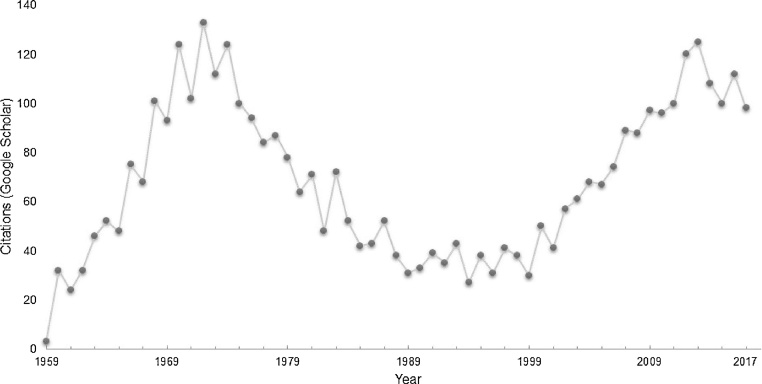
Citations of Adams 1959 [26] description of agar overlay methods, over time. Values are Google Scholar cataloged citations for each year, as reported on December 3rd, 2017.

Nearly 100 years since their first description, plaque assays hold fast as a mainstay in laboratories working with bacterial viruses [[29], [30]] and the agar overlay based approaches continue to represent the gold standard for determining titers and purifying viral strains. The quantitative relationship between viral particles and macroscopic plaques, though not necessarily 1:1, allows simple agar overlay plating techniques to provide consistent estimates of viral titer in stocks of unknown concentration. While these estimates are often lower than those made by flow cytometry or microscopy based methods, the agar overlay plaque assay is the only method that can be readily used to measure infectivity [31]. The continued publication of novel optimizations to the agar overlay to improve efficiency [32] and plaque detectability [[33], [34]], to tailor methods for specific host-virus systems [[35], [36]], and to reduce bias against certain groups of viruses, such as the jumbo viruses [37], speaks to the power and widespread use of this method. Indeed, the number of citations over time of the Adams 1959 [26] description of the method is suggestive of a recent resurgence of interest in cultivation based study of bacterial viruses (Fig. 4).

### Microreview of additional considerations relevant to agar overlays

We close by briefly highlighting additional methodological aspects that are known or expected to impact the nature, number, and diversity of viruses recovered using agar overlay approaches and by providing references to related studies. For overviews as well as detailed guides to methods for working with bacterial viruses the reader is referred to the 1959 vol by Adams [26], as well as to the three recently produced volumes of “Bacteriophages – Methods and Protocols” [[38], [39], [40]]. Importantly, attention towards broad evaluation of impacts of operational approaches and ecological relevance of cultivation conditions is likely to yield the greatest rewards in terms of new diversity; a consideration perhaps best exemplified by the late recognition of the existence of giant viruses [41], which are lost in the 0.2 um pre-filtration step commonly applied to viral samples to remove cells. Incorporation of modifications to address such aspects will likely represent fruitful avenues for discovery of novel viruses from well-studied systems.

• Media Composition of bacterial growth media, bottom agar, and top agar, can affect host expression profiles, virion decay rates and conformations, and the dynamics of plaque formation.
  ○ *Oxygen radicals in media*: Autoclaving media containing agar has been shown to reduce growth of microorganisms [42] through production of reactive oxygen species such as hydrogen peroxide [43], and to result in decreased plaque size in agar overlays [36]. Consider preparing components of the media separately [44] or using microwave-based sterilization [45].
  ○ *Percentage top agar*: Reducing agar concentration in top agar has been shown to allow recovery of large viruses that otherwise do not form visible plaques [37], and has been suggested to facilitate increased recovery of viruses dependent on host motility for infection [46].
  ○ *Treatments and additives to facilitate plaque visualization*: Detection of plaques has been shown in some systems to be improved by treatment of host lawns with contrast-increasing tetrazolium salts [34], and by inclusion of additives that increase the size and clarity of plaques, for example antiobiotics [[47], [48]], glycerol [48], and glycine [[36], [47], [48]].
  ○ *Components necessary for infection to occur:* Presence of salts and divalent cations, especially Ca++ and Mg++, have been shown to play a role in attachment, genome penetration [[49], [50]], and replication [51] of some viruses, with needs for specific cations and salt concentrations varying by virus [52]. Amino acids have also been shown to be important to infection, for example tryptophan has been shown to play a role in inducing changes in the conformation of tail fibers that is necessary to allow virus-host interactions in some strains of T4 [[53], [54], [55]]; notably, this facilitation is also inhibited by indole, a bacterial metabolic product of tryptophan [[56], [56]]. Components of the media also affect bacterial expression and thus the availability of virus-specific receptors on the cell surface.
  ○ *Media selection*: As host-virus interactions that occur in one media formulation may not be observed in another, it is recommended that media selection and formulation be guided by specific study aims rather than assumed to be universal for any one group of bacteria. Exploration of media compositions closely mimicking the features of the milieus where the host and virus interact are likely to yield more diverse isolates and more application relevant findings, in the cases of diversity studies and engineered deployment, respectively.
• Hosts The strains, growth state, and density of hosts at plating will affect the number, diversity, and host range of viruses that can infect and replicate, as well as the size and morphotypes of the resulting plaques.
  • *Host strain selection*: Closely related hosts of the same species often show differing susceptibilities to viruses, an observation put to extensive medical use historically in the phage-typing of pathogens [57]. Within a set of closely related bacteria, some strains may however serve as “indicator strains”, being more broadly susceptible [58], whereas others may enrich for specific groups of viruses, such as those that are dependent on carriage of specific plasmids [59].
  • *Multiple-host approaches*: Sequential exposure to different hosts in serial agar overlays has been shown to increase recovery of polyvalent viruses [60], and mixed-host agar overlays have also been used to isolate viruses infecting multiple hosts [61]. Mixed host overlays have also been employed in the study of lysogeny [62], where low concentrations of hosts carrying resident prophages (lysogens) are mixed in overlay with a background of sensitive indicator hosts and exposed to inductants to yield plaques.
  • *Host growth state and density at plating*: Host growth state and density at time of incorporation into agar overlay can impact plaque formation and detection [36].
• Plating and incubation conditions
  • *Plating and incubation temperatures*: Incubation temperatures bear on both virion stability and host expression profiles [[63], [64]], it is therefore important to consider ecological or application relevance when preparing and incubating agar overlays. Virus-host systems that are particularly heat sensitive may benefit from use of low-melting temperature top agar.
  • *Aerobic or anaerobic conditions*: For hosts that are capable of growing under both aerobic and anaerobic conditions, such as *Escherichia coli*, it may be found that different groups of viruses form plaques under each condition.
  • *Time holding plates for plaque development*: Incubation of agar overlays for extended periods of time has been shown to increase recovery of specific groups of viruses [14].
• Negative controls It is recommended that for each host strain being plated with added virus, an additional host-only overlay also be prepared. This allows for detection of cases where hosts form plaques by autoinduction of resident prophages; as the formation of such autoinduction plaques can be highly sensitive to growth and plating conditions it should not be assumed that a plating from one experiment will be representative of subsequent platings.

### Additional resources

The protocols described here will be made available through the open access protocols repository and forum, protocols.io, under the following names:

• “Tube-free Agar Overlays: rapid plaque assays with fewer steps and materials”
• “Molten Streaking for Singles: rapid tube-free serial purification of viruses”
• “Archiving Plaques: saving virus purification for later”
