# Supplementary material for: Streamlining standard bacteriophage methods for higher throughput
Source: MethodsX. 2018 Jan 31;5:159–72. doi: 10.1016/j.mex.2018.01.007 (PMC6318102; doi:10.1016/j.mex.2018.01.007)
Supplement: Supplementary file 1 [file mmc1.docx]

**Supplementary Material:**

Supplementary File 1. Plaque Archive Spreadsheet example
